# Supplementary material for: Causal Relationship between Aging and Anorexia Nervosa: A White-Matter-Microstructure-Mediated Mendelian Randomization Analysis
Source: Biomedicines. 2024 Aug 16;12(8):1874. doi: 10.3390/biomedicines12081874 (PMC11351342; doi:10.3390/biomedicines12081874)
Supplement: Supplementary file 1 [file biomedicines-12-01874-s001.zip › Supplementary Figure/Figure S9/Leaveoneout plot of MR analysis for ubm-b-1957 to AN.pdf]

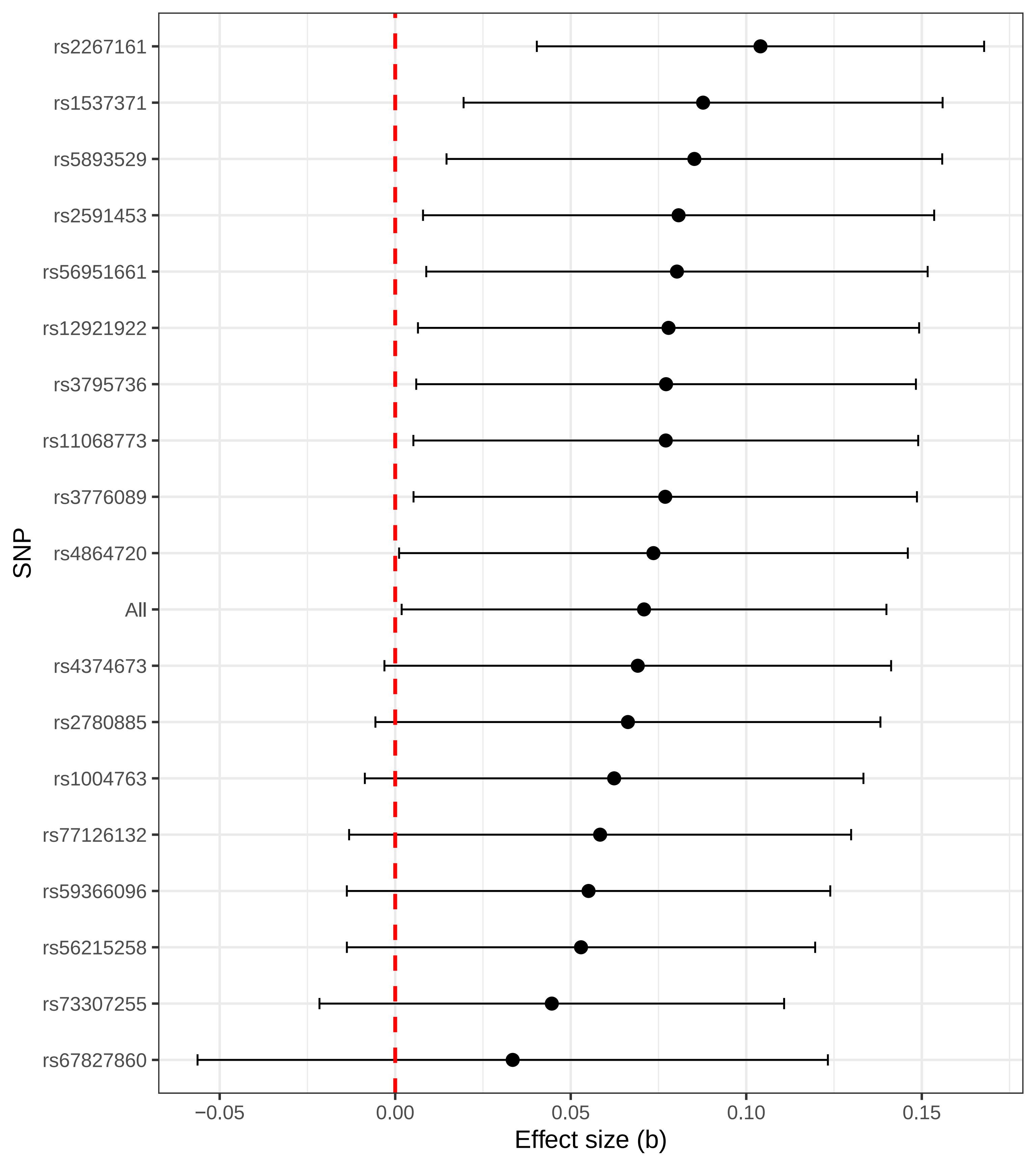

rs2267161
rs1537371
rs5893529
rs2591453
rs56951661
rs12921922
rs3795736
rs11068773
rs3776089
rs4864720
All
rs4374673
rs2780885
rs1004763
rs77126132
rs59366096
rs56215258
rs73307255
rs67827860
SNP
−0.05
0.00
0.05
0.10
0.15
Effect size (b)
